# The efficacy and safety of riluzole for neurodegenerative movement disorders: a systematic review with meta-analysis

**DOI:** 10.1080/10717544.2017.1413446

**Published:** 2017-12-10

**Authors:** Jia Liu, Lu-Ning Wang

**Affiliations:** ^a^ Department of Neurology, Xuanwu Hospital, Capital Medical University Beijing China; ^b^ Department of Geriatric Neurology, Chinese PLA General Hospital Beijing China

**Keywords:** Riluzole, systematic review, meta-analysis, Huntington disease, parkinsonism

## Abstract

Neurodegenerative movement disorders mainly include Parkinson’s disease, atypical parkinsonisms, Huntington disease, and hereditary ataxia. Riluzole is the only drug approved by the US Food and Drug Administration for amyotrophic lateral sclerosis. The neuroprotective effects of riluzole have been observed in experimental models of neurodegenerative movement disorders. In this paper, we aimed to systematically analyze the efficacy and safety of riluzole for patients with neurodegenerative movement disorder. We searched the electronic databases such as PubMed, EMBASE, CINAHL, Cochrane Library and China National Knowledge Infrastructure until June 2017 for the eligible randomized controlled trials, as well as the unpublished and ongoing trials. For continuous data, we calculated standardized mean differences with 95% confidence intervals if studies did not use the same scales to measure outcomes. For dichotomous data, we calculated risk differences if a trial reported no adverse events or dropouts. We pooled the results using a random-effects model. We included nine studies with 1320 patients with neurodegenerative movement disorders, which compared riluzole with placebo. No significant difference was found in the number of participants with adverse events but with motor improvement in hereditary ataxia. There were only two studies focusing on neuroprotective effect. Riluzole is well-tolerated in the patients with neurodegenerative movement disorders. Riluzole seems to be promising for patients with hereditary ataxia in symptomatic effect, which needs to be further confirmed by well-designed studies in the future. Moreover, it makes sense to design long-term study focusing on neuroprotective effect of riluzole in disease-modifying.

## Introduction

Neurodegenerative movement disorders include Parkinson’s disease (PD), atypical parkinsonisms, Huntington disease (HD), and hereditary ataxia, which are characterized by neuron loss and gliosis with clinical motor symptoms (Liu & Wang, 2014). Non-motor symptoms are probably seen in the course of diseases, such as hyposmia, dementia, and autonomic dysfunction. Sporadic PD and atypical parkinsonisms are usually age-related and attributed to tauopathy [progressive supranuclear palsy (PSP) and corticobasal degeneration (CBD)] or synucleinopathy [PD and multiple system atrophy (MSA)] (Dickson, 2012). While HD and hereditary ataxia are hereditary polyglutamine (PolyQ) diseases with early-onset (Katsuno et al., 2014). At present, neurodegeneration and diseases progression cannot be completely prevented by any interventions. The current clinical therapy is mainly focused on relieving symptoms (e.g. hypokinesia, hyperkinesia, or ataxia) and neuroprotective effects (Tsou et al., 2009; Liu et al., 2014; Liu & Wang, 2017).

Traditionally, antiparkinson medications e.g. levidopa and dopamine agonists are used to treat hypokinesia, while dopamine antagonists can release hyperkinesia. However, these therapies are not always effective in controlling symptoms (Connolly & Lang, 2014; Jankovic, 2016; Coppen & Roos, 2017). For instance, levodopa-induced dyskinesias and motor fluctuations can be seen in the advanced stage of PD patients (Aquino & Fox, 2015). Riluzole, an antiglutamatergic agent, is the only drug approved by the US Food and Drug Administration for therapy in amyotrophic lateral sclerosis (ALS). The neuroprotective effects of riluzole have been observed in experimental models of PD, MSA, and HD (Douhou et al., 2002; Schiefer et al., 2002, 2005). Concerning the motor improvement, riluzole reduces motor disturbance in MSA animal models, and may prevent levodopa-induced dyskinesias in PD patients (Merims et al., 1999; Scherfler et al., 2005). In HD patients, riluzole can improve the symptoms and reduce chorea scores with protecting gray matter volume loss and increasing the production of neurotrophins (Bonelli & Hofmann, 2007; Squitieri et al., 2009).

Currently little effective pharmacological treatment is available for patients with neurodegenerative movement disorders. Riluzole might improve motor function and have potential neuroprotective effects in these patients. Some clinical randomized controlled trials (RCTs) have already been conducted (Jankovic & Hunter, 2002; Huntington Study Group, 2003; Braz et al., 2004; Bara-Jimenez et al., 2006; Seppi et al., 2006; Landwehrmeyer et al., 2007; Bensimon et al., 2009; Ristori et al., 2010; Romano et al., 2015). However, there is no systematic review in the current peer-reviewed literature focusing on this topic. In this review, we aimed to systematically analyze the efficacy and safety of riluzole for patients with neurodegenerative movement disorders.

## Methods

We searched the electronic databases such as PubMed, EMBASE, CINAHL, Cochrane Library, and CNKI (China National Knowledge Infrastructure) until June 2017 for all the relevant trials. For unpublished and ongoing trials, we searched the US National Institute of Health clinical trial site (http://www.clinicaltrials.gov/) and the World Health Organization International Clinical Trials Registry Platform (http://www.who.int/ictrp/en/). We also used Science Citation Index Cited Reference Search for forward tracking of important articles, as well as reference lists of relevant reviews and retrieved articles. The key words for search included: (2-Amino-6-trifluoromethoxybenzothiazole OR Riluzole OR Rilutek) AND (Parkinson’s disease OR Multiple system atrophy OR Progressive supranuclear palsy OR Corticobasal degeneration OR Dementia with Lewy bodies OR Huntington’s disease OR ataxia) and their Chinese equivalents. There were no language limitations. We only included RCTs with either parallel or cross-over design. Two authors (LJ and WL) independently evaluated and included the eligible trials. For continuous data, we calculated the mean differences (MDs) with 95% confidence intervals (CIs) or standardized mean differences (SMDs) if studies did not use the same scales to measure outcomes. For dichotomous data, we calculated the risk ratios (RRs) with 95% CIs. If a trial (or group within a trial) reported no adverse events or dropouts, we calculated the risk differences (RDs) instead of the RRs. When there was more than one experimental group in the study, we combined all relevant experimental groups of the study into a single group. Concerning missing standard deviations for changes from baseline, we calculated them with CIs, standard errors, *t* or *p* values, according to the principles provided in Cochrane handbook (Higgins & Green, 2011). We pooled the results using a random-effects model. When there was significant clinical heterogeneity, we gave a descriptive summary of the results. We planned to use funnel plots to examine potential publication bias if more than 10 trials were involved in meta-analysis. Sensitivity analysis was carried out where it was necessary.

## Results

**Figure 1.**
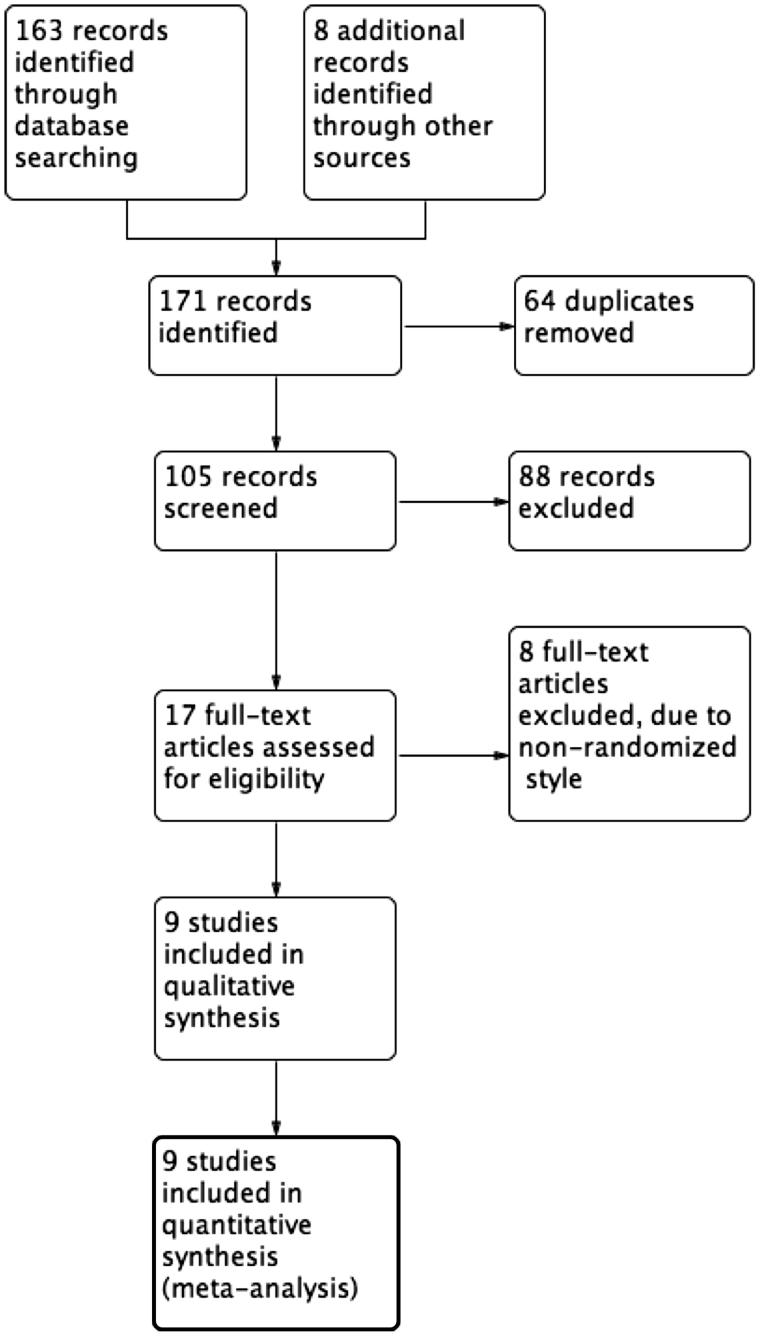
Study flow diagram.

We identified a total of 105 references from the electronic database searches after excluding duplicates (Figure 1). After screening of titles and abstracts, we obtained the full papers of 17 studies and assessed them for eligibility. According to the inclusion criteria, we included nine studies with 1528 randomized patients (Jankovic & Hunter, 2002; Huntington Study Group, 2003; Braz et al., 2004; Bara-Jimenez et al., 2006; Seppi et al., 2006; Landwehrmeyer et al., 2007; Bensimon et al., 2009; Ristori et al., 2010; Romano et al., 2015). The details of included studies were provided in Table 1.

**Table 1.** Baseline characteristics of included studies.

Study	Study design	No. of randomized	Subjects	Intervention	Risk of bias^a^
Bara-Jimenez et al. (2006)	Parallel, 3-stage	15	Subjects with PD	Randomly assigned into riluzole or placebo alone, in a 4:1 ratio. After a 1-week placebo run-in, riluzole were given 1 week at 50 mg bid and then 1 week at 100 mg bid) . The remaining patients received placebo throughout the 3 weeks of study	L,L,L,L,L,L
Bensimon et al. (2009)	Parallel, multicenter	767	Clinically probable PSP and MSA	Stratified for diseases (MSA or PSP) randomly assigned into riluzole (50-200 mg/day) or placebo as ratio 1:1 for 3 years	L,L,L,L,L,L
Braz et al. (2004)	Parallel	16	PD with dyskinesia induced by levodopa	Randomly assigned into riluzole (50 mg bid) or placebo as ratio 1:1 for 7 consecutive days	U,U,U,L,L,L
Jankovic and Hunter (2002)	Parallel	20	Early stage PD	Randomly assigned into riluzole (50 mg bid) or placebo as ratio 1:1 for 6 months	U,U,U,U,L,L
Seppi et al. (2006)	Crossover	10	Clinically probable MSA	Riluzole (100 mg bid) and placebo for 4 weeks each with a 4-week washout period	U,U,U,U,L,L
Huntington Study Group (2003)	Parallel, multicenter	63	Confirmed HD	Randomly assigned into receiving placebo, riluzole 100 mg/day, or riluzole 200 mg/day for 8 weeks	L,L,L,L,L,L
Landwehrmeyer et al. (2007)	Parallel, multicenter	537	Confirmed HD	Randomly assigned in a 2:1 ratio to riluzole (50mg twice daily) or placebo for 3 years	L,U,L,L,L,L
Ristori et al. (2010)	Parallel, multicenter	40	Cerebellar ataxia	Randomly assigned to riluzole (100 mg/day) or placebo for 8 weeks	L,U,L,L,L,L
Romano et al. (2015)	Parallel, multicenter	60	Spinocerebellar ataxia or Friedreich’s ataxia	Random assignment to riluzole (50 mg orally, twice daily) or placebo for 12 months	L,L,L,L,L,L

^a^Risk of bias (random sequence generation, allocation concealment, patient blind, assessor blind, drop-out or withdraw, selective report), L: low risk; U: unclear risk; H: high risk; HD: Huntington disease; MSA: multiple system atrophy; PD: Parkinson’s disease; PSP: progressive supranuclear palsy.

### Efficacy in motor symptoms

**Figure 2.**
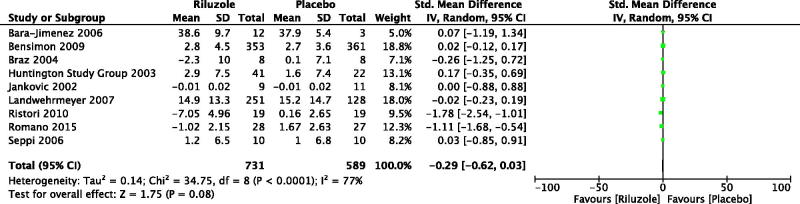
Changes of motor score after the treatment of riluzole versus placebo. Bara-Jimenez et al. (2006): Unified Parkinson's disease Rating Scale (UPDRS) motor. Baseline scores were not provided, however, none of the differences between riluzole and placebo baseline scores were significant. Therefore, we compared the scores at the endpoint. Bensimon et al. (2009): Short Motor Disability Scale. Braz et al. (2004): UPDRS motor. We combined the scores of ON and OFF state as two groups for each intervention. Huntington Study Group (2003): Unified Huntington’s Disease Rating Scale (UHDRS) motor. Jankovic and Hunter (2002): Movement Time (MT) Index score. It was calculated as the averaged performance seconds/count) across the Single Button Index MT, Single Button Wrist MT, and Alternate Button MT tests over three right- and three left-handed trials. Landwehrmeyer et al. (2007): UHDRS motor. Ristori et al. (2010): International Cooperative Ataxia Rating Scale. Romano et al. (2015): the Scale for the Assessment and Rating of Ataxia. Seppi et al. (2006): UPDRS motor. For all the scales, the more scores meant the more severe movement disorders.

As a result, we included nine studies with 1320 patients with neurodegenerative movement disorders, which compared riluzole with placebo (Jankovic & Hunter, 2002; Huntington Study Group, 2003; Braz et al., 2004; Bara-Jimenez et al., 2006; Seppi et al., 2006; Landwehrmeyer et al., 2007; Bensimon et al., 2009; Ristori et al., 2010; Romano et al. 2015). By meta-analysis, the change of motor score was SMD −0.29, 95% CI −0.62 to 0.03, *p* = .08; level of heterogeneity Chi^2^ = 34.75, df = 8, *p* < .000, *I* ^2^ = 77% (Figure 2). For PD, three RCTs with 51 patients were included for analysis (Two RCTs were assessed by Unified Parkinson's disease Rating Scale (UPDRS) motor, and one RCT was evaluated by Movement Time Index score) (Jankovic & Hunter, 2002; Braz et al., 2004; Bara-Jimenez et al., 2006). The change of motor score was SMD −0.08, 95% CI −0.66 to 0.51, *p* = .80; level of heterogeneity Chi^2^ = 0.22, df = 2, *p* = .90, *I* ^2^ = 0%. For atypical parkinsonisms, two RCTs with 734 patients with MSA or PSP were synthesized (Two RCTs were assessed by UPDRS motor and Short Motor Disability Scale, respectively) (Seppi et al., 2006; Bensimon et al., 2009). The change of motor score was SMD 0.02, 95% CI −0.12 to 0.17, *p* = .74; level of heterogeneity Chi^2^ = 0.00, df = 1, *p* = .99, *I* ^2^ = 0%. Two RCTs with 442 HD patients were included and analyzed (Both of two RCTs were evaluated by Unified Huntington’s Disease Rating Scale motor) (Huntington Study Group, 2003; Landwehrmeyer et al., 2007). The change of motor score was SMD 0.01, 95% CI −0.19 to 0.20, *p* = .95; level of heterogeneity Chi^2^ = 0.46, df = 1, *p* = .50, *I* ^2^ = 0%. There were two RCTs with 93 patients with hereditary ataxia measured by International Cooperative Ataxia Rating Scale (ICARS) and Scale for the Assessment and Rating of Ataxia, respectively (Ristori et al., 2010; Romano et al., 2015). Significant improvement in motor symptoms was found in riluzole versus placebo. The change of motor score was SMD −1.39, 95% CI −2.04 to −0.74, *p* < .000; level of heterogeneity Chi^2^ = 1.89, df = 1, *p* = .17, *I* ^2^ = 47%.

### Efficacy in neuroprotective effects

For parkinsonism, only one RCT referred to neuroprotective effects (Jankovic & Hunter, 2002). There were 342 participants (45.0%) died during the double-blind period, with no difference between the PSP and the MSA patients [171 participants with PSP (47.2%); 171 participants with MSA (43.0%); by the log-rank test]. 95 survivals of 181 participants in riluzole group and 96 survivals of 181 participants in placebo group for the participants with PSP (RR 0.99 (95% CI 0.81 to 1.20). 109 survivals of 199 participants in riluzole group and 118 survivals of 199 participants in placebo group for the participants with MSA (RR 0.92 (95% CI 0.78 to 1.10). For HD, one RCT with 379 patients reported total functional capacity (TFC) score as the measurement of neuroprotective effects (Landwehrmeyer et al., 2007). The change of TFC score was MD −0.10, 95% CI −0.60 to 0.40, *p* = .69, with no significant difference between riluzole and placebo. No RCT on hereditary ataxia was involved in the outcomes of neuroprotective effects.

### Safety

**Figure 3.**
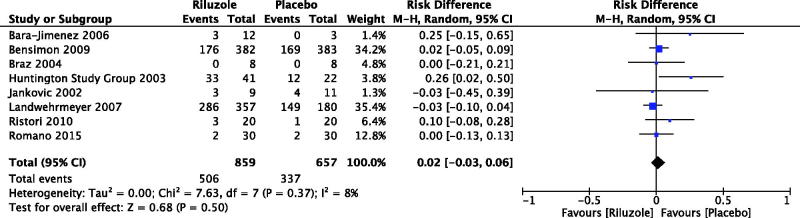
Number of participants with adverse events. Bensimon et al. (2009): The data of serious adverse events but not adverse events were available.

For PD patients, there were three RCTs reported the number of participants with adverse events (Jankovic & Hunter, 2002; Braz et al., 2004; Bara-Jimenez et al., 2006), with no significant between riluzole and placebo (RD 0.04, 95% CI −0.13 to 0.21, *p* = .65; level of heterogeneity Chi^2^ = 1.28, df = 2, *p* = .53, *I* ^2^ = 0%) (Figure 3). Only one RCT discussed serious adverse events but not adverse events in patients with atypical parkinsonisms (RD 0.02, 95% CI −0.05 to 0.09, *p* = .59) (Bensimon et al., 2009). For PolyQ diseases, the number of participants with adverse events was reported by two RCTs in HD patients (RD 0.09, 95% CI −0.19 to 0.37, *p* = .51; level of heterogeneity Chi^2^ = 5.08, df = 1, *p* = .02, *I* ^2^ = 80%) (Huntington Study Group, 2003; Landwehrmeyer et al., 2007), and two RCTs in patients with hereditary ataxia (RD 0.03, 95% CI −0.07 to 0.14, *p* = .54; level of heterogeneity Chi^2^ = 0.80, df = 1, *p* = .37, *I* ^2^ = 0%) (Ristori et al., 2010; Romano et al., 2015). By meta-analysis of eight RCTs (1516 participants), the number of participants with adverse events was RD 0.02, 95% CI −0.03 to 0.06, *p* = .50; level of heterogeneity Chi^2^ = 7.63, df = 7, *p* = .37, *I* ^2^ = 8% (Figure 3) (Jankovic & Hunter, 2002; Huntington Study Group, 2003; Braz et al., 2004; Bara-Jimenez et al., 2006; Landwehrmeyer et al., 2007; Bensimon et al., 2009; Ristori et al., 2010; Romano et al., 2015).

## Discussion

In general, riluzole is well-tolerated in the patients with neurodegenerative movement disorders, no matter in short-term (one year or less) or in long-term (more than one year). Only two RCTs were riluzole in long-term therapy (3 years) in comparison with placebo for atypical parkinsonisms and HD, respectively (Landwehrmeyer et al., 2007; Bensimon et al., 2009). Neither of them found significant neuroprotective effects. Thus, it is reasonable to design the trials of riluzole in long-term for the patients with neurodegenerative movement disorders to evaluate neuroprotective effects in disease-modifying. The outcomes should include survivals, quality of life, and/or other functional capacities. It has been estimated that riluzole remained the only disease-modifying medication available now with a survival advantage of three months for patients with ALS (Jenkins et al., 2014). Moreover, the accompanying symptoms such as pain and cognitive impairment are also required to be determined if they can be improved by riluzole (Corcia & Meininger, 2008).

As the primary focus, symptomatic effects were reported by all the included RCTs. Most of RCTs were with short-term therapy, however, the assessment of motor symptoms was not affected as the condition in neuroprotective effects. Measurement of motor symptoms included UPDRS Motor, Short Motor Disability Scale, Movement Time Index Score, UHDRS Motor, ICARS, and the Scale for the Assessment and Rating of Ataxia. The heterogeneity of outcome design caused troubles in data synthesis and interpretation, although more scores meant the more severe movement disorders for all the measurements. Actually, UPDRS Motor, UHDRS Motor, and ICARS are the most commonly used scales and should be respectively recommended to parkinsonisms, HD as well as hereditary ataxia. Meanwhile, the data should be provided in detail (with the format Mean ± Standard Deviation) at baseline and endpoint, which are applicable to meta-analysis.

The mechanism of action of riluzole is unknown. At clinical concentrations of riluzole, it has been reported that the relevant neural effects included inhibition of persistent Na^+^ current (i.e. inhibition of repetitive firing), potentiation of calcium-dependent K^+^ current, inhibition of neurotransmitter release, and inhibition of fast Na^+^ current (Bellingham, 2011). Moreover, mitochondrial dysfunction plays a vital role at early stage of neurodegeneration. The deficit of energy increases vulnerability to glutamatergic stimulation and aggravates neurodegeneration (Schapira et al., 2014). Neuronal activity in the subthalamic nucleus, involved in projecting to internal segment of globus pallidus via glutamate, is strengthened (Oertel & Schulz, 2016). Augmentation of the synaptic efficacy of striatal ionotropic glutamatergic receptors seems to be correlated to the appearance of dyskinesias (de Bartolomeis et al., 2015). Therefore, the glutamate antagonist riluzole may be beneficial to neurodegeneration.

The participants, interventions, and settings of the studies are comparable with the patients usually treated in clinical practice. Because of small sample size and short duration of riluzole treatment, it is still unknown whether riluzole can alter the natural history of neurodegeneration. Although our search strategy is rigorous with nine RCTS identified, it is possible that certain studies are not identified, e.g. publications that are not in English and not included in any database we searched. Methodology of some included trials is not clearly described, which affects the judgement in quality of evidence. Concerning the unclear data expressed by graphs, there is no additional information available in contacting with the related authors. Moreover, we cannot assess publication bias using funnel plots with insufficient RCTs included.

## Conclusions

Riluzole is well-tolerated in the patients with neurodegenerative movement disorders, no matter in short-term or in long-term. There are insufficient data to support the usage of riluzole in patients with PD, atypical parkinsonisms and HD to improve motor symptoms or disease-modifying. Riluzole seems to be promising for patients with hereditary ataxia in symptomatic effect, which needs to be further confirmed by well-designed studies in the future. Moreover, it makes sense to design long-term study focusing on neuroprotective effect of riluzole in disease-modifying.

## References

[CIT0001] Aquino CC, Fox SH. (2015). Clinical spectrum of levodopa-induced complications. Mov Disord 30:80–9. 25488260 10.1002/mds.26125

[CIT0002] Bara-Jimenez W, Dimitrova TD, Sherzai A, et al (2006). Glutamate release inhibition ineffective in levodopa-induced motor complications. Mov Disord 21:1380–3. 16758479 10.1002/mds.20976

[CIT0003] Bellingham MC. (2011). A review of the neural mechanisms of action and clinical efficiency of riluzole in treating amyotrophic lateral sclerosis: what have we learned in the last decade? CNS Neurosci Ther 17:4–31. 20236142 10.1111/j.1755-5949.2009.00116.x PMC6493865

[CIT0004] Bensimon G, Ludolph A, Agid Y, et al (2009). Riluzole treatment, survival and diagnostic criteria in Parkinson plus disorders: the NNIPPS study. Brain 132:156–71. 19029129 10.1093/brain/awn291 PMC2638696

[CIT0005] Bonelli RM, Hofmann P. (2007). A systematic review of the treatment studies in Huntington’s disease since 1990. Expert Opin Pharmacother 8:141–53. 17257085 10.1517/14656566.8.2.141

[CIT0006] Braz CA, Borges V, Ferraz HB. (2004). Effect of riluzole on dyskinesia and duration of the on state in Parkinson disease patients: a double-blind, placebo-controlled pilot study. Clin Neuropharmacol 27:25–9. 15090933 10.1097/00002826-200401000-00008

[CIT0007] Connolly BS, Lang AE. (2014). Pharmacological treatment of Parkinson disease: a review. JAMA 311:1670–83. 24756517 10.1001/jama.2014.3654

[CIT0008] Coppen EM, Roos RA. (2017). Current pharmacological approaches to reduce chorea in Huntington's disease. Drugs 7:29–46. 10.1007/s40265-016-0670-4 PMC5216093 27988871

[CIT0009] Corcia P, Meininger V. (2008). Management of amyotrophic lateral sclerosis. Drugs 68:1037–48. 18484797 10.2165/00003495-200868080-00003

[CIT0010] de Bartolomeis A, Errico F, Aceto G, et al (2015). D-aspartate dysregulation in Ddo(-/-) mice modulates phencyclidine-induced gene expression changes of postsynaptic density molecules in cortex and striatum. Prog Neuropsychopharmacol Biol Psychiatry 62:35–43. 25979765 10.1016/j.pnpbp.2015.05.003

[CIT0011] Dickson DW. (2012). Parkinson’s disease and parkinsonism: neuropathology. Cold Spring Harb Perspect Med 2:a009258. 22908195 10.1101/cshperspect.a009258 PMC3405828

[CIT0012] Douhou A, Debeir T, Murer MG, et al (2002). Effect of chronic treatment with riluzole on the nigrostriatal dopaminergic system in weaver mutant mice. Exp Neurol 176:247–53. 12093102 10.1006/exnr.2002.7935

[CIT0013] Higgins JPT, Green S (editors). (2011). Cochrane Handbook for Systematic Reviews of Interventions Version 5.1.0 [updated March 2011]. The Cochrane Collaboration. Available from www.cochrane-handbook.org

[CIT0014] Huntington Study Group (2003). Dosage effects of riluzole in Huntington's disease: a multicenter placebo-controlled study. Neurology 61:1551–6. 14663041 10.1212/01.wnl.0000096019.71649.2b

[CIT0015] Jankovic J. (2016). Dopamine depleters in the treatment of hyperkinetic movement disorders. Expert Opin Pharmacother 17:2461–70. 27819145 10.1080/14656566.2016.1258063

[CIT0016] Jankovic J, Hunter C. (2002). A double-blind, placebo-controlled and longitudinal study of riluzole in early Parkinson's disease. Parkinsonism Relat Disord 8:271–6. 12039422 10.1016/s1353-8020(01)00040-2

[CIT0017] Jenkins TM, Hollinger H, McDermott CJ. (2014). The evidence for symptomatic treatments in amyotrophic lateral sclerosis. Curr Opin Neurol 27:524–31. 25110934 10.1097/WCO.0000000000000135

[CIT0018] Katsuno M, Watanabe H, Yamamoto M, et al (2014). Potential therapeutic targets in polyglutamine-mediated diseases. Expert Rev Neurother 14:1215–28. 25190502 10.1586/14737175.2014.956727

[CIT0019] Landwehrmeyer GB, Dubois B, de Yébenes JG, et al (2007). Riluzole in Huntington's disease: a 3-year, randomized controlled study. Ann Neurol 62:262–72. 17702031 10.1002/ana.21181

[CIT0020] Liu J, Bader B, Danek A. (2014). Neuroacanthocytosis in china: a review of published reports. Tremor Other Hyperkinet Mov (NY) 4:248. 10.7916/D8Q23XDX PMC4219110 25374764

[CIT0022] Liu J, Wang LN. (2014). Mitochondrial enhancement for neurodegenerative movement disorders: a systematic review of trials involving creatine, coenzyme Q10, idebenone and mitoquinone. CNS Drugs 28:63–8. 24242074 10.1007/s40263-013-0124-4

[CIT0021] Liu J, Wang LN. (2017). Hyperkinesia in ancient China: perspectives and prescriptions. CNS Spectr 22:251–3. 28571592 10.1017/S1092852916000560

[CIT0023] Merims D, Ziv I, Djaldetti R, et al (1999). Riluzole for levodopa-induced dyskinesias in advanced Parkinson’s disease. Lancet 353:1764–5. 10347995 10.1016/S0140-6736(99)00120-8

[CIT0024] Oertel W, Schulz JB. (2016). Current and experimental treatments of Parkinson disease: a guide for neuroscientists. J Neurochem 139:325–37. 27577098 10.1111/jnc.13750

[CIT0025] Ristori G, Romano S, Visconti A, et al (2010). Riluzole in cerebellar ataxia: a randomized, double-blind, placebo-controlled pilot trial. Neurology 74:839–45. 20211908 10.1212/WNL.0b013e3181d31e23

[CIT0026] Romano S, Coarelli G, Marcotulli C, et al (2015). Riluzole in patients with hereditary cerebellar ataxia: a randomised, double-blind, placebo-controlled trial. Lancet Neurol 14:985–91. 26321318 10.1016/S1474-4422(15)00201-X

[CIT0027] Schapira AH, Olanow CW, Greenamyre JT, et al (2014). Slowing of neurodegeneration in Parkinson’s disease and Huntington’s disease: future therapeutic perspectives. Lancet 384:545–55. 24954676 10.1016/S0140-6736(14)61010-2

[CIT0028] Scherfler C, Sather T, Diguet E, et al (2005). Riluzole improves motor deficits and attenuates loss of striatal neurons in a sequential double lesion rat model of striatonigral degeneration (Parkinson variant of multiple system atrophy). J Neural Transm 112:1025–33. 15583958 10.1007/s00702-004-0245-5

[CIT0029] Schiefer J, Landwehrmeyer GB, Lüesse HG, et al (2002). Riluzole prolongs survival time and alters nuclear inclusion formation in a transgenic mouse model of Huntington’s disease. Mov Disord 17:748–57. 12210870 10.1002/mds.10229

[CIT0030] Seppi K, Peralta C, Diem-Zangerl A, et al (2006). Placebo-controlled trial of riluzole in multiple system atrophy. Eur J Neurol 13:1146–8. 16987170 10.1111/j.1468-1331.2006.01452.x

[CIT0031] Squitieri F, Orobello S, Cannella M, et al (2009). Riluzole protects Huntington disease patients from brain glucose hypometabolism and grey matter volume loss and increases production of neurotrophins. Eur J Nucl Med Mol Imaging 36:1113–20. 19280185 10.1007/s00259-009-1103-3

[CIT0032] Tsou AY, Friedman LS, Wilson RB, et al (2009). Pharmacotherapy for Friedreich ataxia. CNS Drugs 23:213 23. 19320530 10.2165/00023210-200923030-00003

